# Supplementary material for: A Novel Lactobacillus acidophilus Strain Isolated from a 2-Month-Old Shiba Inu: In Vitro Probiotic Evaluation Safety Assessment in Mice and Whole-Genome Sequencing Analysis
Source: Microorganisms. 2025 Sep 8;13(9):2095. doi: 10.3390/microorganisms13092095 (PMC12472203; doi:10.3390/microorganisms13092095)
Supplement: Supplementary file 1 [file microorganisms-13-02095-s001.zip › microorganisms-3776320-supplementary.pdf]

Supplementary material

Table S1. Inhibition test results of *L. acidophilus* L1.

**Items**	**MRSA**	***S.typhimurium***	***K.pneumoniae***	***S.aureus***	***E.coli***
CFS	25.0±2.5	26.8±0.3	22.5±1.5	29.3±0.8	20.2±0.3
BS	24.3±1.2	26.5±0.5	21.8±0.5	25.6±0.5	18.5±0.3
BP	—	—	—	—	—
MRS broth	—	—	—	—	—

Table S2. Prediction of antibiotic resistance genes in *L. acidophilus* L1.

Gene ID	Location	Class	Resistance gene	phenotype	Resistance Mechanism	Accession no	Identity (%)	Coverage (%)
gene1087	Chromosome	Lincosamide	lnu(C)	Lincomycin	Enzymatic inactivation	AY928180	98.79	100
gene0057	Chromosome	Tetracycline	tet(W)	Doxycycline, Tetracycline, Minocycline	Target protection	FN396364	99.06	100

Table S3. Prediction of virulence factors in *L. acidophilus* L1.

Gene ID	Location	VFDB ID	Vfs	Species	VFcategory	Related genes	Identity (%)	Coverage (%)
gene1596	Chromosome	VFG000964 (gb|WP_010922799)	Hyaluronic acid capsule (VF0244)	Streptococcus pyogenes M1 GAS	Immune modulation	hasC	67.8	97
gene1374	Chromosome	VFG046465 (gb|WP_003028672)	EF-Tu (VF0460)	Francisella tularensis subsp. tularensis SCHU S4	Adherence	tufA	68.7	99.2
gene0754	Chromosome	VFG037100 (gb|WP_010980745)	MsrAB (VF0456)	Neisseria meningitidis MC58	Stress survival	msrA/B pilB	65.6	89

gene1530	Chromosome	VFG000077 (gb|NP_465991)	ClpP (VF0074)	Listeria monocytogenes EGD-e	Stress survival	clpP	69.6	98.5
gene1889	Chromosome	VFG047039 (gb|WP_003018140)	LPS (VF0542)	Francisella tularensis subsp. tularensis SCHU S4	Immune modulation	wbtL	62.9	99.3
gene1920	Chromosome	VFG000964 (gb|WP_010922799)	Hyaluronic acid capsule (VF0244)	Streptococcus pyogenes M1 GAS	Immune modulation	hasC	67.6	99.3
gene0424	Chromosome	VFG012095 (gb|WP_003435012)	GroEL (VF0594)	Clostridium difficile 630	Adherence	groEL	64	99.6
